# Reconsidering Animal Models of Major Depressive Disorder in the Elderly

**DOI:** 10.3389/fnagi.2016.00188

**Published:** 2016-08-08

**Authors:** Shigenobu Toda, Yoshio Iguchi, Ziqiao Lin, Hiromi Nishikawa, Tatsuya Nagasawa, Hirotaka Watanabe, Yoshio Minabe

**Affiliations:** ^1^Department of Psychiatry and Neurobiology, Kanazawa UniversityKanazawa, Japan; ^2^Research Center for Child Mental Development, Kanazawa UniversityKanazawa, Japan; ^3^Hokuriku Dementia Professional Physician Training PlanKanazawa, Japan; ^4^Department of Molecular Genetics, Institute of Biomedical Sciences, Fukushima Medical UniversityFukushima, Japan; ^5^Department of Physiology, Keio University School of MedicineTokyo, Japan

**Keywords:** major depressive disorder, elderly people, dementia, neurodegeneration, animal model

## Introduction

Major depressive disorder (MDD) is a common psychiatric illness with high morbidity that poses a huge burden to healthcare systems worldwide. According to the World Health Organization, the lifetime prevalence of MDD is approximately 3–17% globally (Richards, 2011). However, after major, but limited, success with selective serotonin reuptake inhibitors (SSRIs) or serotonin–noradrenaline reuptake inhibitors, few promising therapeutic approaches have been developed from preclinical studies using current animal models of MDD, despite intensive research involving laborious methods and substantial costs.

Traditionally, most research using rodent models of MDD has been conducted using relatively young adult animals, aged approximately 8 weeks, to avoid any involvement of aging-related biological factors. The major rationale of this strategy is based on epidemiological findings that the occurrence of MDD displays a robust peak in young adults rather than in the elderly (Jorm, 2000; Copeland et al., 2004; Blazer and Hybels, 2005). Further, the elderly may have aging-related factors rendering this group an inappropriate model of “genuine” MDD. A critical question is whether this strategy is indeed appropriate. Despite the consistent findings of a lower lifetime prevalence rate of MDD in the elderly than in young adults, there remain persistent doubts regarding the underdiagnosis of MDD in the elderly (Hoertel et al., 2013). It has been heavily argued that patients diagnosed with MDD in youth will often be rediagnosed with a bipolar disorder (Lish et al., 1994; Leonpacher et al., 2015). Thus, a considerable number of cases of MDD in young adults may be misdiagnosed. In addition, MDD in the elderly has distinct biological/environmental backgrounds and consequences compared with that in young adults.

In this opinion article, we primarily shed light on the significance of MDD in the elderly (known as geriatric MDD or late-onset depression) and problems associated with the methodology of preclinical studies undertaken to investigate the characteristics and treatment of this disorder.

## Influences of aging on physiological functions

First, it is widely accepted that functional changes in motor or cognitive functions in the elderly, including those related to learning and memory, are attributed to aging-induced physiological changes (Mora et al., 2007; Grady, 2012; Limdenberger, 2014). For example, aging of the dopaminergic system is one of the major factors responsible for motor disturbances in the elderly. However, the patients with Parkinson's disease may demonstrate dysregulated habits and learning abilities before manifesting motor disturbance as a result of dorsal striatum damage (Hadj-Bouziane et al., 2013). This type of inconspicuous dysfunction may be sufficient to affect the ability of the elderly to perform normal daily activities, which consequentially generates frustration, exhaustion, and compromised self-esteem. Meanwhile, the effect of aging on the serotonergic or noradrenergic system, which plays a pivotal role in the pathophysiology of MDD (Blier and El Mansari, 2013; Fakhoury, 2015), remains to be elucidated in rodents and humans, although a decline in serotonin transport in the elderly both in human and non-human primates (Kakiuchi et al., 2001; Yamamoto et al., 2002) may result not only in mood instability but also in being short-tempered or easily giving up in the elderly (Miyazaki et al., 2012). Besides neurotransmitters, aging-induced decreases in neurotropic factors, such as brain-derived neurotropic factor (BDNF), which is essential for neuronal plasticity, may also be involved in cognitive dysfunction (Burke and Barnes, 2006; Tapia-Arancibia et al., 2008).

Current major hypotheses to explain the molecular mechanisms underlying MDD, such as a deficit of BDNF (Duman and Monteggia, 2006), dopamine (Nestler and Carlezon, 2006; Dreher et al., 2008), serotonin, or stress-induced attenuation of neurogenesis (Lazarov et al., 2010; Hamilton et al., 2013). However, these potential mechanisms are more suitable for aged than younger brains because these alterations commonly become eminent during the natural course of aging. Other aging-related macro/microstructural changes, such as vascular changes, weakened immune/cytokine responses, compromised redox status, a loss of dendritic spines, and reduced neuronal plasticity, may also account for the pathophysiology of MDD (Burke and Barnes, 2006; Lucin and Wyss-Coray, 2009; Currais and Maher, 2013; Gutchess, 2014). All these physiological systems maintain resilience as long as they function appropriately. However, once malfunction begins, it may result in the loss of resilience, namely an increased prevalence of damaging symptoms such as cognitive impairment (Butters et al., 2004), pseudo-dementia, and delusions (Alexopoulos, 2005), which occur less often in young adults. These symptoms are related to the higher rate of recurrence of MDD in the elderly (Maeshima et al., 2012) and complicate the treatment of these patients. Executive functions and decision-making abilities may be similarly affected (Buckner, 2004; Breton et al., 2015; Samanez-Larkin and Knutson, 2015).

## Causal relationship between MDD and dementia in the elderly

Second, researchers has recently argued the possibility of MDD as a precursor or risk factor for Parkinson's disease (Fang et al., 2010; Inoue et al., 2010; Shen et al., 2013; Gustafsson et al., 2015), mild cognitive impairment (Panza et al., 2010; Richard et al., 2013; Snowden et al., 2015), dementia with Lewy bodies (Boot et al., 2013), vascular dementia (Barnes et al., 2012; Diniz et al., 2013; Taylor et al., 2013), Alzheimer's disease (AD; Green et al., 2003; Ownby et al., 2006; Geerlings et al., 2008; Barnes et al., 2012; Diniz et al., 2013), or general aging–related cognitive disorders (Byers and Yaffe, 2011; da Silva et al., 2013; Sibille, 2013). As mentioned before, MDD in the elderly often accompanies pseudo-dementia, which alleviates when MDD is attenuated but not completely in many cases (Alexopoulos, 2005), implying a continuum of deterioration from MDD to dementia in the elderly. In support of this concept, MDD in the elderly often involves a greater degree of severe brain atrophy and/or white matter hyperintensity than that in the age-matched healthy controls (Herrmann et al., 2008; Dotson et al., 2009; Köhler et al., 2010; Tham et al., 2011; Geerlings et al., 2013; Ribeiz et al., 2013; Khundakar and Thomas, 2014).

Given that depression in the elderly is a precursor of dementia, depression should always precede the onset of dementia, and concomitantly coexist at the initial or transitional stage of dementia. However, given the considerable prevalence of depression without cognitive impairment in the elderly (Pellegrino et al., 2013), in some cases, MDD may boost, rather than prelude, dementia. At this point, whether a history of depression is a precursor for dementia remains an issue of debate.

Even in case that MDD is not the precursor for neurodegenerative disorders, the process of neurodegeneration, in turn, may cause vulnerability against MDD from a very early stage. For example, Lewy bodies or senile plaques appear in the brain at a relatively early point in life, while the consequence could be subclinical in terms of cognitive function. However, the appearance of plaques, albeit a subthreshold of the manifestation of motor or cognition impairment, may provide sufficient vulnerability to stress for triggering MDD by gradually damaging physiological resilience (Jagust, 2013). In this scenario, MDD is not a precursor but rather a sinister overture of dementia. The increase of Aβ levels in the cerebrospinal fluid (CSF) in patients with MDD supports this idea (Osorio et al., 2014). Intriguingly, SSRIs, which are not effective against the core symptoms of AD, were found to decrease Aβ levels in CSF in both healthy individuals and AD model mice (Sheline et al., 2014), suggesting that AD pathology is reversible, at least to some extent, and is not sufficient to develop dementia by itself.

## Further promising relationships between stress and dementia

Meanwhile, the causal relationship between chronic stress and dementia has been repeatedly supported by many preclinical studies (Csernansky et al., 2006; Sotiropoulos et al., 2008; Dong and Csernansky, 2009; Rissman, 2009). For example, chronic stress or glucocorticoid augmentation induces tau pathology in rat brain (Catania et al., 2009; Carroll et al., 2011), as well as cognitive impairment in rats (Rissman et al., 2007, 2012; Sotiropoulos et al., 2011). Corticotropin releasing factor accelerates Aβ production in AD models (Dong et al., 2012). In addition, chronic mild stress accelerates the onset and progression of AD-like phenotypes in Tg2576 mice (Cuadrado-Tejedor et al., 2012). Moreover, stress not only induces posttraumatic stress disorder-like phenotypes but also more easily elevates Aβ levels in AD model animals compared with wild-type animals (Justice et al., 2015). However, in these studies, MDD-like symptoms or treatment-resistance to antidepressants were not assessed. Thus, at this point, it is still inconclusive whether they are the models of dementia which includes depression as one of the initial symptoms or pseudo-dementia in elderly MDD. Meanwhile, clinical studies have not yet addressed the effect of psychological stress on dementia. On the contrary, it is well known that cognitive enrichment or social engagement can delay the onset of dementia, even with the same load of AD pathology (Stern, 2006; Fratiglioni and Wang, 2007), which further supports the idea that MDD may boost dementia as an additional factor by affecting these positive factors.

## Other factors that exaggerate MDD in the elderly

**Figure 1:**
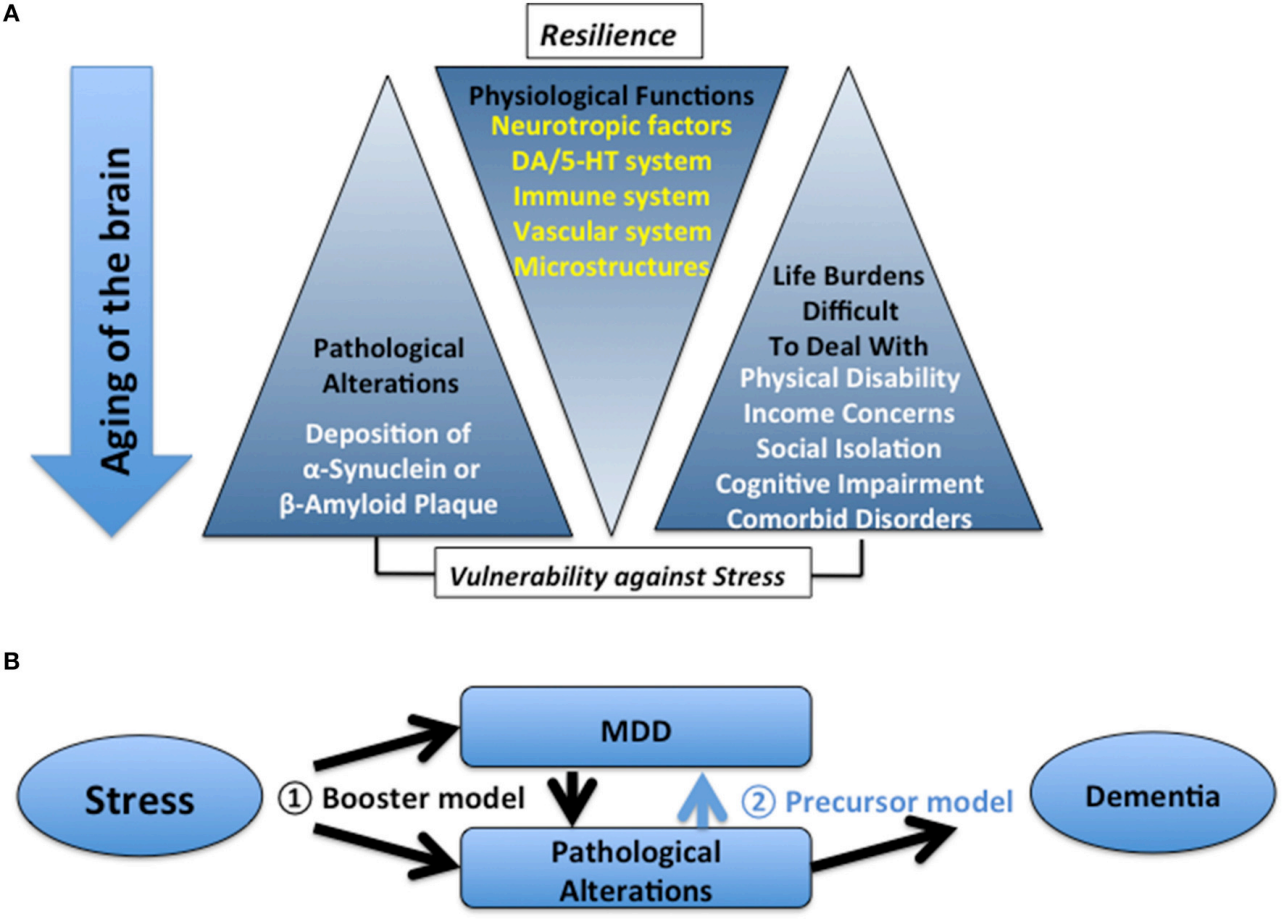
**(A)** Relationship between aging-related factors in patients with dementia and MDD. **(B)** Two models for understanding the causal relationship between MDD and dementia.

Other factors that are not directly connected to the aging of the brain, such as the presence of comorbid illnesses and corresponding pharmacological treatment for these illnesses, social isolation, or spousal loss, could predispose, or deteriorate MDD in the elderly. Overall, in the elderly, vulnerability to stress is more profound. Thus, it is highly likely that depression-like phenotypes may be more easily inducible by various stressors in the elderly than in young adults (summarized in Figure 1A). In addition, because of the abovementioned multiple complicated factors, MDD in the elderly demonstrates diverse individual differences both in symptoms and responses to antidepressants, resulting in treatment failure and higher recurrence in the elderly than in young adults (Reynolds et al., 1999).

## Essential preclinical studies for elucidating the molecular pathophysiology underlying MDD in the elderly

Taken together, two current models regarding the relationship between MDD, and dementia in the elderly are summarized (Figure 1B). In the first model, stress induces dementia-related pathology and MDD independently and the co-existence of the two will result in dementia, indicating that the presence of MDD promotes the development of dementia (i.e., booster model). In this sense, the full remission of MDD in the elderly may be more important to prevent future dementia. In the second model, stress induces dementia-related pathology to a point sufficient for the development of dementia; MDD appears as a prodromal symptom of dementia (i.e., precursor model). In this case, to halt the ensuing symptoms, it is important to elucidate the molecular mechanisms underlying the development and manifestation of depression (Gareri et al., 2002; Krishnan, 2002; Alexopoulos and Kelley, 2009; Naismith et al., 2012).

We propose a methodology to verify which model is correct. To verify the “booster” model, it is important to determine whether AD-related pathology is sufficient to induce MDD-like symptoms without other stress in rodents (Llorens-Martin et al., 2011). In turn, it would be intriguing to address the effect of subclinical AD-related pathology on the vulnerability to stress in animal models of dementia before the exhibition of robust cognitive or motor dysfunction at certain ages by exposure to other types of stress that may induce MDD. Relatively young or middle-aged animals are appropriate for this purpose and are used in the most current preclinical studies. On the other hand, to verify the second model, resistance to treatment with antidepressants and persistence of these pathological alterations, cognitive impairments, which are the hallmarks of dementia, and MDD-like symptoms need to be fully addressed in stress-induced AD models. Given that MDD is a part of dementia as this scenario, it is expected that there will be very fewer resilience cases in the elderly than in young adults. In addition, other types of “pure” psychological stressors that evoke MDD-like phenotypes, such as social defeat (Berton et al., 2006), should be examined to reproduce similar consequences. Hence, such studies require aged rodents.

Our proposed studies may appear to be an amalgamation of various interdependent aging-related factors rather than a “genuine” model of MDD. However, they may mimic both actual clinical situations and the brain states of patients at the onset of MDD better than conventional animal models of MDD. These approaches may allow us to conclude a causal relationship between MDD and dementia in the elderly. In addition, they may help to develop novel treatment regimens to not only alleviate the symptoms of MDD but also prevent the onset of dementia in the elderly.

## Author contributions

ST, YI, LZ, HN, TN, HW, and YM designed and wrote the manuscript.

### Conflict of interest statement

The authors declare that the research was conducted in the absence of any commercial or financial relationships that could be construed as a potential conflict of interest.

## Abbreviations

Aβ: amyloid-β
AD: Alzheimer's disease
BDNF: brain-derived neurotropic factor
CSF: cerebrospinal fluid
MDD: major depressive disorder
SSRI: selective serotonin reuptake inhibitor.
